# Bridging global and local topology in whole-brain networks using the network statistic jackknife

**DOI:** 10.1162/netn_a_00109

**Published:** 2020-02-01

**Authors:** Teague R. Henry, Kelly A. Duffy, Marc D. Rudolph, Mary Beth Nebel, Stewart H. Mostofsky, Jessica R. Cohen

**Affiliations:** Department of Psychology and Neuroscience, University of North Carolina at Chapel Hill, Chapel Hill, NC, USA; Department of Psychology and Neuroscience, University of North Carolina at Chapel Hill, Chapel Hill, NC, USA; Department of Psychology and Neuroscience, University of North Carolina at Chapel Hill, Chapel Hill, NC, USA; Center for Neurodevelopmental and Imaging Research, Kennedy Krieger Institute, Baltimore, MD, USA; Department of Neurology, Johns Hopkins School of Medicine, Baltimore, MD, USA; Center for Neurodevelopmental and Imaging Research, Kennedy Krieger Institute, Baltimore, MD, USA; Department of Neurology, Johns Hopkins School of Medicine, Baltimore, MD, USA; Department of Psychiatry and Behavioral Science, Johns Hopkins School of Medicine, Baltimore, MD, USA; Department of Psychology and Neuroscience, University of North Carolina at Chapel Hill, Chapel Hill, NC, USA

**Keywords:** Network, Statistics, fMRI, Functional connectivity, Graph theory, Whole-brain analysis, Jackknife

## Abstract

Whole-brain network analysis is commonly used to investigate the topology of the brain using a variety of neuroimaging modalities. This approach is notable for its applicability to a large number of domains, such as understanding how brain network organization relates to cognition and behavior and examining disrupted brain network organization in disease. A benefit to this approach is the ability to summarize overall brain network organization with a single metric (e.g., global efficiency). However, important local differences in network structure might exist without any corresponding observable differences in global topology, making a whole-brain analysis strategy unlikely to detect relevant local findings. Conversely, using local network metrics can identify local differences, but are not directly informative of differences in global topology. Here, we propose the *network statistic (NS) jackknife framework*, a simulated lesioning method that combines the utility of global network analysis strategies with the ability to detect relevant local differences in network structure. We evaluate the NS jackknife framework with a simulation study and an empirical example comparing global efficiency in children with attention-deficit/hyperactivity disorder (ADHD) and typically developing (TD) children. The NS jackknife framework has been implemented in a public, open-source R package, *netjack*, available at https://cran.r-project.org/package=netjack.

## INTRODUCTION

Describing the brain as a network, an approach termed *network neuroscience* (Bassett & Sporns, 2017), is a powerful method that allows regions distributed across the entire brain to be incorporated into a single model describing overall brain topology. This method has provided insights into whole-brain disruption in neurological and psychiatric diseases (Fornito, Bullmore, & Zalesky, 2017; Fornito, Zalesky, & Breakspear, 2015), the dynamic processes underlying cognition (Cohen & D’Esposito, 2016; Kucyi, Tambini, Sadaghiani, Keilholz, & Cohen, 2018), and changes across development (Baum et al., 2017; Grayson & Fair, 2017). Researchers using a network neuroscience framework can draw upon the rich methodological and statistical literature in graph theory and network analysis, grounding the approach in a well-established mathematical framework.

However, whole-brain analysis methods, as commonly applied in network neuroscience, often fail at what Hallquist and Hillary (Hallquist & Hillary, 2018) refer to as “matching theory to scale.” This refers to the fact that whole-brain network analysis methods operate at a global resolution, while relevant differences might be very localized. In other words, there may be differences at a local scale (i.e., within a single subnetwork) that would be missed when calculating a single, whole-brain summary measure that describes global topology. Conversely, observed global differences in topology could be driven by a specific local difference (i.e., an observed overall difference in network integration could be driven by a difference limited to a single subnetwork). This mismatch in resolution, therefore, could obscure meaningful differences in network structure. While local network statistics, such as participation coefficient of a single node, can be used to identify local differences of interest, these local statistics do not directly assess a localized difference in global topology, and as such are not ideal for matching a global theory to a local scale. In the present manuscript, we develop the *network statistic (NS) jackknife* as a general method to localize global network statistics across a variety of network metrics and brain features of interest, thus allowing researchers to better address this resolution issue.

The NS jackknife method (available for use at https://cran.r-project.org/package=netjack) proposed here allows researchers to identify specific brain regions, connections, or subnetworks (i.e., *local* features) that drive *global* network analysis results or, conversely, that are not detected by global network analysis. By doing so, our method can detect local differences in network structure using the same summary metrics that a global analysis would use, as opposed to relying on local metrics that do not explicitly link to the global metrics in question. This approach bridges the gap between a global and a local scope of analysis by allowing researchers to apply global analysis strategies in a way that reveals differences at local scopes, even when those differences would not be detected with the global analysis method. We evaluate the performance of the NS jackknife with a simulation study and demonstrate the utility of this method with an empirical example comparing resting-state brain network organization in children with attention-deficit/hyperactivity disorder (ADHD) and age-matched typically developing (TD) children.

### Global Versus Local Topology

There are many network statistics that can be used to analyze the global topology of a network; for simplicity we focus on two widely used network statistics as examples. First, we use *modularity*, or a measure of a network’s segregation into multiple communities (or subnetworks; Newman, 2006). Second, we use *global efficiency,* a statistic that assesses the overall integration of a network (Latora & Marchiori, 2001). These two statistics have led to great insight regarding brain organization, in particular for supporting the hypothesis that the balance between network segregation (dense, within-subnetwork connectivity) and network integration (communication across distinct subnetworks) is critical for cognition (for reviews and canonical articles, see the following: Cohen & D’Esposito, 2016; Shine & Poldrack, 2017; Sporns, 2013; Sporns, Chialvo, Kaiser, & Hilgetag, 2004).

However, a key issue for whole-brain analysis is its poor detection of local differences in topology. For example, if a whole-brain analysis produces differences in overall network topology between groups or conditions, it does not provide insight into *where* in the brain these differences might arise. Further, if a whole-brain analysis produces no differences in overall network topology, that does not preclude the existence of important local differences. This property of whole-brain network analysis is less than ideal as it ignores local features, which can include specific brain regions, specific connections between brain regions, or specific subnetworks. A local topological difference arising from one region/connection/subnetwork versus another could have vastly different implications for empirical inferences and resultant clinical applications.

There are several existing graph theory metrics that operate on a local scale. For example, *within-module degree* indicates which nodes are more central to their communities, and *participation coefficient* indicates which nodes connect different communities (Guimerà & Nunes Amaral, 2005). Additionally, there are a variety of statistical models for identifying nodes and other network features of importance. One of the most widely used is the network-based statistic method (NBS; Zalesky, Fornito, & Bullmore, 2010), which identifies differences between groups by examining maximally different sets of edges. The degree-based statistic method of Yoo and colleagues (Yoo et al., 2017) is more focused than that of the NBS method, and identifies highly central nodes that are related to a hypothesis of interest. Another recent approach, that of screen filtering (Meskaldji et al., 2015), allows researchers to analyze group differences in nodal and edge-wise metrics; however, this method as currently implemented is restricted to nodal, edge-wise or subnetwork statistics as it relies on correcting a collection of statistical tests. All of these methods allow researchers to assess local differences between groups and thus are powerful tools for understanding different features of network organization, but none of these methods assess local drivers of global topological properties of a network. For example, a common method for examining group differences in local modular structure is examining differences in participation coefficient for individual nodes or subsets of nodes. While nodes that exhibit a participation coefficient difference might correspond to nodes that are relevant to differences in *global* modularity, this is by no means assured. Local metrics and tests can be related to global metrics (i.e., participation coefficient can be related to whole-brain modularity), but these relations are not one-to-one, and a difference in local properties does not necessarily imply a difference in global properties. Similarly, previous statistical models that identify network elements of importance do not identify these elements based on their relative contribution to global topology, but rather based on their impacts on local topology. These properties of local metrics and existing local statistical models suggest a need for a framework that can assess differences in global topology and directly link these global differences to local structures.

The disconnect that arises between the global and the local scope can be due to specificity, in which whole-brain network results are driven by specific connections, regions, or subnetworks; or to equifinality, in which strikingly different network configurations can lead to the same whole-brain summary metric values. These two concepts can be related, as equifinality can be caused by specific differences in local topology that do not lead to differences in summary metrics describing global topology. The NS jackknife, proposed here, provides a solution to these issues. This statistical approach allows researchers to simultaneously analyze differences in global topology and localize these differences to specific brain regions, connections, or subnetworks.

### Network Specificity and Equifinality

*Specificity* is the network principle whereby differences in observed whole-brain or whole-network topology can be driven by specific differences in local topology, as opposed to globally distributed differences. A whole-brain network difference can be specific if, for example, one group of interest has greater connectivity within one specific functional subnetwork than a different group, and this increase in connectivity is substantial enough that it alone drives a significant difference in global modularity. Conversely, a whole-brain network difference can be nonspecific if the difference in topology is due to a diffuse pattern of local differences. For example, if one group has higher connectivity between all pairs of subnetworks, this can result in a difference in global efficiency that cannot be localized to any given subset of networks. One key issue with whole-brain network analysis is that it is not capable of determining whether an observed topological difference is specific or nonspecific. This property has been termed the *theory-to-scale issue* (Hallquist & Hillary, 2018).

**Figure 1.**
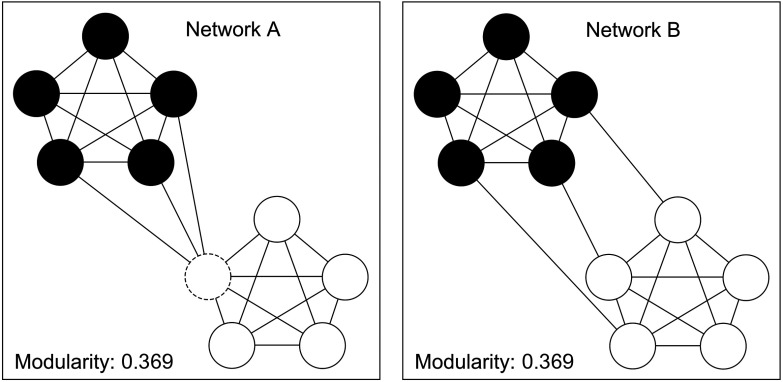
Two networks with the same value of modularity yet very different inter-subnetwork patterns of connectivity. Note that Network A contains a single connector hub, or a node that links two communities (dashed circle), while Network B does not.

This lack of specificity is complicated by the fact that, when calculating various summary statistics for brain networks, typical approaches in network neuroscience can produce the same network statistics for different network configurations. *Equifinality*, a term borrowed from system theory (Bertalanffy, 1950), is the network principle whereby the same result can be achieved by many different means. Here, we use it to emphasize the fact that the same inference can result from different global network configurations. For example, Figure 1 shows two networks, each consisting of two subnetworks. The difference between these networks is in how these subnetworks are connected. In Network A, the communities are connected via a *hub node* (dotted circle), while for Network B the subnetworks are connected more diffusely. Whole-network modularity of the two networks in Figure 1 is identical, yet this result masks the fact that the local structure of the inter-subnetwork connections in the two networks is quite different. This demonstrates the possible disconnect between whole-brain findings (i.e., global scope) and specific local differences in topology (i.e., local scope).

Specific versus nonspecific whole-brain findings occur with regularity in neuroimaging research. For example, individuals suffering from Alzheimer’s disease exhibit reductions in default mode network connectivity as well as global loss of a small-world network structure (Pievani, de Haan, Wu, Seeley, & Frisoni, 2011). This analysis of whole-brain small-world structure differentiates patients with Alzheimer’s disease from healthy control participants but provides no information as to the role default mode network connectivity plays in that global finding. The NS jackknife method has the ability to quantify whether reductions of whole-brain small-world structure in Alzheimer’s disease are driven by the default mode network alone, are widely distributed across all subnetworks in the brain, or are driven by a small number of subnetworks.

These specific local differences with prominent global differences found in Alzheimer’s disease can be contrasted with the finding that individuals with autism spectrum disorder (ASD) tend to exhibit increased whole-brain integration, as assessed with global efficiency, as compared with healthy control participants (Lewis, Theilmann, Townsend, & Evans, 2013; Rudie & Dapretto, 2013). While other research has shown specific differences between individuals with ASD and healthy control participants, for example within the salience network (Di Martino et al., 2009), the finding of increased functional integration appears be a result of more diffuse and weaker functional connections distributed across the entire brain, and thus a whole-brain phenomenon (Roine et al., 2015; Rudie & Dapretto, 2013). While a whole-brain network analysis would accurately detect this group difference, it would give no indication whether the difference was due to a widely distributed whole-brain difference or driven by specific subnetworks or connections, a conclusion that could be made with use of the NS jackknife. These examples emphasize that whole-brain network analysis strategies are designed to examine a global scope but lack the ability to determine the specificity of any given finding (Hallquist & Hillary, 2018).

### Localization of Whole-Brain Analysis Using the NS Jackknife

The NS jackknife localizes whole-brain network analyses by iteratively removing elements of the network, such as edges, nodes, or subnetworks, and recalculating the target network statistic without those elements. By sweeping through the whole network, as well as assessing the impact of the removal across subjects, the NS jackknife creates an empirical distribution of network element effects, allowing a researcher to localize specific features of brain network organization that contribute most strongly to a given whole-brain network statistic. The NS jackknife was inspired by the traditional jackknife estimator used extensively in nonparametric statistics and can be considered a virtual lesioning technique.We are using the term “jackknife” not only because the NS jackknife relies on the leave-one-out approach of the traditional jackknife, but also because like the traditional jackknife, the NS jackknife is a general statistical technique that can be applied to any question that entails localizing a global network statistic.

A jackknife in statistics is a resampling technique used to adjust for outliers using a leave-one-out approach (Efron, 1982; Tukey, 1958). This procedure removes a single observation, such as a single subject’s data, from a sample, recalculates the effect estimate, then adds the removed observation back into the sample and removes another; it iterates through the sample in this fashion. In standard sampling situations, in which each observation is the behavioral data of a single individual, this method is useful for detecting potential outliers. Jackknife techniques have been previously used in network science to assess the overall robustness of a network statistic to node removal (Snijders & Borgatti, 1999). However, previous uses of this technique have focused on single instances of a network (i.e., one social network) and on obtaining estimates of a network statistic, rather than on assessing the importance of particular network structures in a set of multiple networks (i.e., multiple brain networks).

While a standard jackknife procedure removes individual subjects in turn from an analysis, we are proposing here to remove elements of a graph, such as nodes, edges, or subnetworks. Here for the sake of example, we focus on the removal of entire functional subnetworks, or tightly interconnected sets of regions of interest (ROIs) thought to underlie various aspects of cognition, such as the default mode network (Greicius, Krasnow, Reiss, & Menon, 2003) or the salience network (Seeley et al., 2007). Once a subnetwork is removed, the network statistic is recalculated, and the procedure continues with the removal of another subnetwork. Specific versions of this procedure have been used in previous neuroimaging research (e.g., Achard, Salvador, Whitcher, Suckling, & Bullmore, 2006; Henry, Dichter, & Gates, 2018); we develop a general framework here. Critically, while our NS jackknife estimator is based on previous work in simulated lesioning, and at its core is a simulated lesioning method (Achard et al., 2006; Alstott, Breakspear, Hagmann, Cammoun, & Sporns, 2009; Crucitti, Latora, Marchiori, & Rapisarda, 2004), it is focused on localization of network effects rather than the overall robustness of a network. More specifically, while robustness focuses on understanding the overall resiliency of a network to attack, localization attempts to understand the impact of the removal of specific elements on the network. Robustness and localization are highly related, and the NS jackknife differs from previous simulated lesioning methods mainly in interpretation and in its specific focus on understanding group differences. Additionally, while the focus here is on testing group differences without subject- or network-level covariates, the NS jackknife framework can be extended to more complex models that can account for covariates and other design factors. In this sense, the NS jackknife is an extension of the simulated lesioning technique into a general testing framework for localizing global network statistics. Finally, we provide an easily accessible implementation of the NS jackknife in the *netjack* package.

### The NS Jackknife

To describe the NS jackknife in more precise terms, let $\left\{G_{1},\ldots G_{n}\right\}$ be the observed networks of *n* subjects. Let *f*(⋅)be the functional form of some network statistic, such as modularity, and let *k* be the indicator of a feature of interest (such as a subnetwork), taken from some set *K* of size |*K*|. $G_{i\left[-k\right]}$ is the network of individual *i* with feature *k* removed. More concretely, if we were analyzing functional connectivity networks, then *G*_*i*_ would be the whole-brain functional connectivity network of individual *i*, and $G_{i\left[-DMN\right]}$ would be the functional connectivity network of individual *i* with all the nodes of the default mode network removed.

The set of original network statistics is *f*(⋅) applied to all *G*_1_, … *G*_*n*_ , and this *n*-length vector is denoted as $\hat{f}$. The jackknife estimate with regard to feature *k* is then $f\left(\cdot \right)$ applied to all $G_{1\left[-k\right]},\ldots ,G_{n\left[-k\right]}$; this length *n* vector is denoted as ${\hat{f}}_{-k}$. Finally, the difference between the original network statistic and the jackknife estimator with regard to structure *k* is denoted as ${\hat{d}}_{-k}$.

Applying this procedure to the |*K*| features of interest (e.g., all functional subnetworks) leads to $\left|K\right|$ jackknife estimate vectors and $\left|K\right|$ difference vectors for each subject. If subjects are divided between Groups A and B, we denote the estimate and difference vectors as ${\hat{f}}_{-k}^{\left(A\right)},{\hat{f}}_{-k}^{\left(B\right)}$ and ${\hat{d}}_{-k}^{\left(A\right)}$, ${\hat{d}}_{-k}^{\left(B\right)}$.

There are several methodological considerations when using the NS jackknife. The first is that network statistics are a priori dependent on network size and density (van Wijk, Stam, & Daffertshofer, 2010); comparing network statistics across different subsets of networks of different sizes can lead to spurious findings. In the NS jackknife framework, this issue is handled by comparing networks of the same size (as in the group test) or changes in network statistics (as in the differential impact test).

Additionally, the NS jackknife produces a series of *dependent* multiple comparisons. In traditional jackknife settings, observations removed at each iteration are independent of all other observations. However, in the NS jackknife the removal of individual elements from every subject’s network and the subsequent reestimation of network statistics results in a series of dependent statistical tests. Because of the dependent nature of these tests, and the risk for inflation because of multiple comparisons, the Benjamani-Hochberg (BH) procedure is an appropriate false discovery rate (FDR) correction for multiple comparisons (Benjamini & Hochberg, 1995). Other multiple comparisons corrections that allow for dependent tests, such as the Benjamani-Yekutieli (BY; Benjamini & Yekutieli, 2001) correction, are also appropriate, while the Bonferroni correction would not be, as it does not account for the presence of dependent tests. If the network components of interest are nodes or edges, and the subnetwork membership of these components is known, the screen filtering approach of Meskaldji et al. (2015) provides a way of correcting for multiple comparisons that results in more power to detect effects than the BH or BY corrections, and should be preferentially used.

All analyses and graphical outputs in this manuscript use the *netjack* open source R package (Henry, 2018), a package tailor-made for performing the NS jackknife in a simple to use, customizable fashion.

### Localizing Group Differences and Differential Impact

The primary use of the NS jackknife is to localize global network statistic differences between groups to specific subnetworks, nodes, or edges. Consider the example of comparing the whole-brain modularity for a given set of subnetworks between two clinical groups. In addition to quantifying differences across the groups in global modularity using standard network statistics, the NS jackknife allows for two additional questions of substantive interest:

1.  **Group differences:** Given the removal of a subnetwork, are the two groups’ mean global values significantly different from each other? This corresponds to a hypothesis of the following:
  $$E\left[{\hat{f}}_{-k}^{\left(A\right)}\right]\neq E\left[{\hat{f}}_{-k}^{\left(B\right)}\right].$$
2.  **Differential impact:** Is there a differential impact of the removal of a subnetwork between the two groups? This corresponds to a hypothesis of the following:
  $$E\left[{\hat{d}}_{-k}^{\left(A\right)}\right]\neq E\left[{\hat{d}}_{-k}^{\left(B\right)}\right].$$

We illustrate the use of these two tests in the following simulated example.

**Figure 2.**
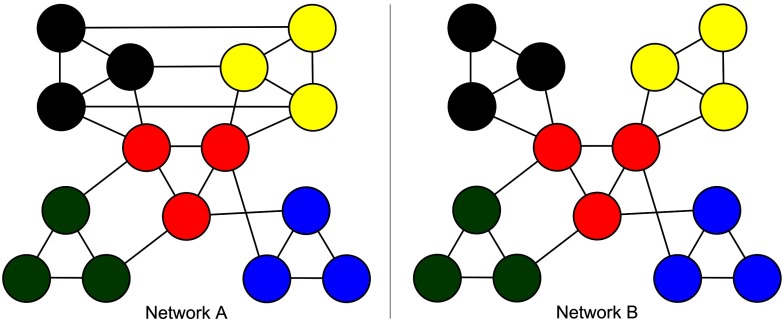
Two modular networks. The red module acts as a central hub for both networks, while in Network A the black and yellow modules are directly interconnected.

*Data generation*. For Group A, the sample of networks was generated using a stochastic block model for binary undirected networks (Nowicki & Snijders, 2001), with probabilities set to create the observed community structure (0.9 within, 0.5 between). The sample contained 10 “subjects.” For Group B, data generation used the same procedure and the same base probabilities (0.9 within, 0.5 between), with additional connections between the yellow and black groups set to 0.5 probability (see Figure 2). Group B, like Group A, contained 10 “subjects.”

*Analysis*. The NS jackknife was used to quantify the effect of the removal of each of the five subnetworks on each subject’s overall network modularity. For the group differences analysis, a two-sample *t* test was conducted to determine whether the updated modularity due to the removal of any given subnetwork was significantly different across groups. For the differential impact analysis, a two-sample *t* test was conducted on the *difference* in modularity due to the removal of any given subnetwork to determine whether there was a differential impact across the groups in removing each subnetwork. For the differential impact analysis, *p* values were corrected for five tests using the BH correction for dependent multiple comparisons. Group difference test *p* values are uncorrected.

**Figure 3.**
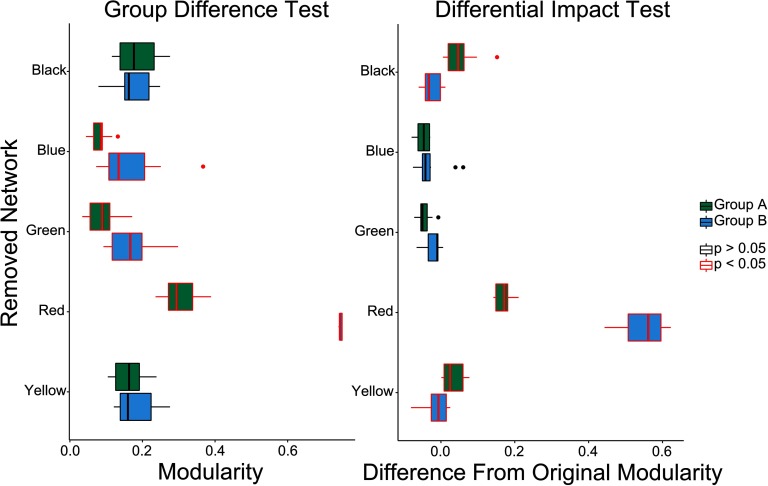
Group differences (left figure) and differential impact (right figure) of subnetwork removal on modularity of Network A (in blue) and Network B (in green) from Figure 2. Central line is the median, box is the interquartile range (IQR), and whiskers represent 1.5 * *IQR* . Points represent values greater than 2 *SD* from the mean. Both group difference and differential impact tests suggest that the red network is significantly more centrally integrated in Group B than in Group A. The differential impact tests additionally show that removal of the black or yellow network causes more of an increase in modularity for Group A than Group B, suggesting that these subnetworks are more integrated into the whole network in Group A.

*Results*. Whole-brain modularity was significantly higher in Group B than in Group A ( *t*(13.41) = 2.9288, *p* < 0.05 ; average modularity 0.1974 and 0.1332, respectively). For the group differences analysis, the whole-brain group difference (B > A) remained after removal of the blue, green, or red subnetworks, while removal of the black or yellow subnetworks resulted in no group difference in modularity (Figure 3, left panel). For the differential impact analysis, removal of the black and yellow subnetworks resulted in an increased change in modularity for Group A as compared with Group B. Removal of the red subnetwork resulted in an increased change in modularity for Group B as compared with Group A (although modularity increased for both groups), and removal of the blue and green subnetworks resulted in a comparable (and near-zero) change in modularity across the groups (Figure 3, right panel).

*Interpretation*. The red subnetwork was highly integrated in both Group A and Group B; however, its removal led to less of an impact on modularity in Group A than Group B. The black and yellow subnetworks were more integrated into the rest of the network in Group A than Group B.

Comparing group differences with differential impact analysis permits one to directly assess the impact that local differences can have on global findings. To summarize, the removal of the red subnetwork corresponded to a significant group difference as well as a significant differential impact, suggesting that its removal led to the networks becoming more different from each other. In contrast, the removal of the yellow or black subnetworks led to significant differential impact but no significant group differences, indicating that the removal of either of these subnetworks led to the networks becoming more similar to one another.

### Simulation Study: Specificity and Sensitivity of the NS Jackknife

A simulation study assessing the specificity and sensitivity of the NS jackknife to the magnitude of group differences, sample size, and network size was conducted. Results show that NS jackknife has good sensitivity to detect small group differences in the generating model at moderate sample sizes (50 subjects per group) and larger networks (250 nodes) and shows excellent sensitivity for larger group differences across multiple conditions. In a condition in which groups had different whole-network statistics that were driven by specific local differences, NS jackknife detected differences in differential impact across all subnetworks (Condition 4, Supplementary Figure 5), and an examination of effect sizes correctly identified which subnetworks had the most impact on the network statistic (Supplementary Figure 6). In all conditions tested, network size was an important predictor of power, with larger networks demonstrating greater power to detect small effects.

One key conclusion from this simulation study regards the interpretation of NS jackknife results when the tested groups are significantly different in their whole-brain statistic. In this case, the NS jackknife tends to detect differences upon the removal of all the network elements being jackknifed on. In this case, two routes of interpretation are viable. The first is to examine the effect sizes of differences to determine which component of the network contributes most to the differences between the groups (i.e., which has the largest effect size). The second is to determine whether any subnetwork was not found to have a significant differential impact, in which case it can be concluded that those subnetworks were not significant contributors to the observed group difference.

For full details of this simulation study, see the Supporting Information.

### Empirical Example: Reduced Integration of Subnetworks in ADHD

This empirical example is a proof of concept demonstrating how the use of the NS jackknife provides important information about differences in functional brain network organization in children with ADHD and TD children. ADHD is a neurodevelopmental disorder characterized by difficulty with sustaining attention, as well as excessive impulsive or hyperactive behavior (American Psychiatric Association, 2013). Existing research indicates that whole-brain functional and structural topology are disrupted in individuals with ADHD, as are specific local differences in connectivity (for reviews, see the following: Cao, Shu, Cao, Wang, & He, 2014; Henry & Cohen, 2019). Individuals with ADHD exhibit higher whole-brain functional segregation than healthy control participants (Lin et al., 2014; Wang et al., 2009). In terms of specific local differences, there appears to be disruptions in networks such as the default mode, frontoparietal, attention, visual, and motor subnetworks (Castellanos & Proal, 2012). Given these findings at the global and local level, a comparison of individuals with ADHD and healthy control participants is a useful test case for the NS jackknife. In the following analyses, we examine global efficiency (Latora & Marchiori, 2001) of resting-state functional MRI scans in children with ADHD as compared with TD children. Global efficiency is a measure of functional integration. Previous studies have mixed findings with regard to differences in global efficiency, either showing that individuals with ADHD have slightly lower global efficiency than healthy control participants (Lin et al., 2014) or no differences in global efficiency (Wang et al., 2009). In this example, we attempt to localize potential differences in global efficiency to specific functional subnetworks in an effort to learn more about these inconsistencies in the prior literature.

## METHODS

### Sample Characteristics and Data Acquisition

Participants included 80 children with ADHD between 8 and 12 years of age (male *N* = 55 and female *N* = 25 ) and 208 age-matched TD children (male *N* = 150 and female *N* = 58 ). For details on study inclusion criteria, see Barber and colleagues (2015).

All data were collected at the Kennedy Krieger Institute (Baltimore, MD) using a Philips 3T Achieva MRI scanner. High-resolution T1-weighted 3D MPRAGE images were acquired with the following parameters: repetition time (TR) = 8.05 ms, echo time (TE) = 3.76 ms, flip angle = 8°, matrix 256 × 256, field of view (FOV) = 200 mm, and slice thickness 1 mm. For resting-state data, 21 participants had 128 T2*-weighed echoplanar images (EPIs), while 167 participants had 156, both collected with the following parameters: TR = 2.5 s, TE = 30 ms, flip angle = 70°, matrix 96 × 96, and FOV = 256 mm.

### Preprocessing

The resting-state images were preprocessed using FMRIPREP version 1.0.7 (Esteban et al., 2018), a Nipype-based tool (Gorgolewski et al., 2011). Each T1-weighted volume was corrected for intensity nonuniformity using N4BiasFieldCorrection v2.1.0 (Tustison et al., 2010) and skull-stripped using antsBrainExtraction.sh v2.1.0 (using the OASIS template). Brain surfaces were reconstructed using recon-all from FreeSurfer v6.0.1 (Dale, Fischl, & Sereno, 1999), and the brain mask estimated previously was refined with a custom variation of the method to reconcile ANTs-derived and FreeSurfer-derived segmentations of the cortical graymatter of Mindboggle (Klein et al., 2017). Spatial normalization to the ICBM 152 Nonlinear Asymmetric template version 2009c (Fonov, Evans, McKinstry, Almli, & Collins, 2009) was performed through nonlinear registration with the antsRegistration tool of ANTs v2.1.0 (Avants, Epstein, Grossman, & Gee, 2008), using brain-extracted versions of both the T1-weighted volume and template. Brain tissue segmentation of cerebrospinal fluid (CSF), whitematter (WM), and graymatter (GM) was performed on the brain-extracted T1-weighted image using FAST (Zhang, Brady, & Smith, 2001).

Functional data were slice time corrected using 3dTshift from AFNI v16.2.07 (Cox, 1996) and motion corrected using mcflirt (FSL v5.0.9; Jenkinson, Bannister, Brady, & Smith, 2002). This was followed by coregistration to the corresponding T1-weighted image using boundary-based registration (Greve & Fischl, 2009) with 9 degrees of freedom, using bbregister (FreeSurfer v6.0.1). Motion-correcting transformations, BOLD-to-T1-weighted transformation, and T1- weighted-to-template (MNI) warp were concatenated and applied in a single step using antsApplyTransforms (ANTs v2.1.0) using Lanczos interpolation. Frame-wise displacement (FD) (Power et al., 2013) was calculated for each functional run using the implementation of Nipype.

Many internal operations of FMRIPREP use Nilearn (Abraham et al., 2014), principally within the BOLD-processing workflow. For more details of the pipeline see https://fmriprep.readthedocs.io/en/latest/workflows.html. This description of the preprocessing pipeline was generated at http://fmriprep.readthedocs.io/en/latest/citing.html.

### Motion Processing and Spectral Filtering

Following preprocessing with FMRIPREP, the resting-state scans were corrected for nuisance variables, including motion, WM signal, and CSF signal. This correction proceeded in the following steps. Timepoints that exceeded 0.3mm FD (Power, Barnes, Snyder, Schlaggar, & Petersen, 2012) were flagged, as were the timepoints immediately preceding and following the flagged timepoint. Interpolation across these flagged timepoints was conducted using spectral interpolation (Ciric et al., 2017). Following the spectral interpolation, a high-pass filter at 0.008 MHz was applied to the voxel time series containing the interpolated timepoints. The flagged timepoints were then set to missing for all voxels. A nuisance regression set consisting of the 6 degrees of motion and mean WM and CSF signal, as well as the temporal derivatives, quadratic expansions, and the quadratic expansions of the temporal derivatives of these 8 regressors, was high-pass filtered at 0.008 MHz, as per the suggestion of Ciric, Satterthwaite, and colleagues (Ciric et al., 2017; Satterthwaite et al., 2013). The filtered nuisance regressors were used to regress out nuisance signal from the scrubbed and filtered voxel time series. Finally, subjects that had more than 30% of their data lost because of scrubbing were removed from the analysis, as were subjects who exceeded 0.2 mm mean FD overall. This resulted in a final sample of 34 children with ADHD and 121 TD children, with mean percentage timepoints lost 8% and 10% respectively. This difference in percentage timepoints lost was nonsignificant(*t*(48.423) = −1.47, *p* = 0.14 ). Additionally, the mean FD in the final sample was not significantly different between children with ADHD and TD children (*t*(49.902) = 1.27, *p* = 0.21, ADHD mean = 0.131, TD mean = 0.121).

### ROI Extraction and Network Construction

From the preprocessed and motion-corrected resting-state images, ROI time series were extracted using a commonly used functional brain atlas (Power et al., 2011). This atlas consists of 264 ROIs, 234 of which are classified into 13 functional subnetworks. These functional subnetworks and abbreviations are as follows: auditory (AUD), cerebellar (CER), cingulo-opercular (CO), default mode (DMN), dorsal attention (DAN), frontoparietal (FP), memory (MEM), salience (SAL), somatomotor hand (SM Hand), somatomotor mouth (SM Mouth), subcortical (SUB), ventral attention (VAN), and visual (VIS). From these ROI time series, 234 × 234 correlation matrices were computed. These correlation matrices were then converted to absolute values and thresholded at a correlation magnitude of 0.35 to create binary connectivity networks. The pattern of findings below was consistent for a band of thresholds from 0.25 to 0.4.

### Network Statistics and Jackknife

In this application, we use global efficiency as our network statistic. Global efficiency is defined as the average of the inverse shortest path length between all nodes in the network (Latora & Marchiori, 2001), and can be thought of as a measure of global integration. We applied the jackknife over the 13 functional subnetworks in the functional brain atlas. The two tests previously described were then applied to the jackknifed data. This allowed us to assess (a) whether the removal of a functional subnetwork led to a significant difference in global efficiency between children with ADHD and TD children; and (b) whether the impact of the removal of a functional subnetwork on functional integration was different for children with ADHD as compared with TD children.

### Jackknife Estimate and Group Comparisons

Independent two-sample *t* tests were conducted to compare global efficiency values across the entire sample, and to compare global efficiency values and differential impacts across groups. Differential impact results were corrected for multiple dependent comparisons using the BH correction, as described in the above simulations.

## RESULTS

Group differences in whole-brain global efficiency: There was no group difference in global efficiency for children with ADHD and TD children (*t*(50_3_7) = 0.99, *p* = 0.32; ADHD mean = 0.29, TD mean = 0.28).

Group differences by subnetwork removal: Next, we examined group differences in global efficiency between children with ADHD and TD children after removing each functional subnetwork.

**Figure 4.**
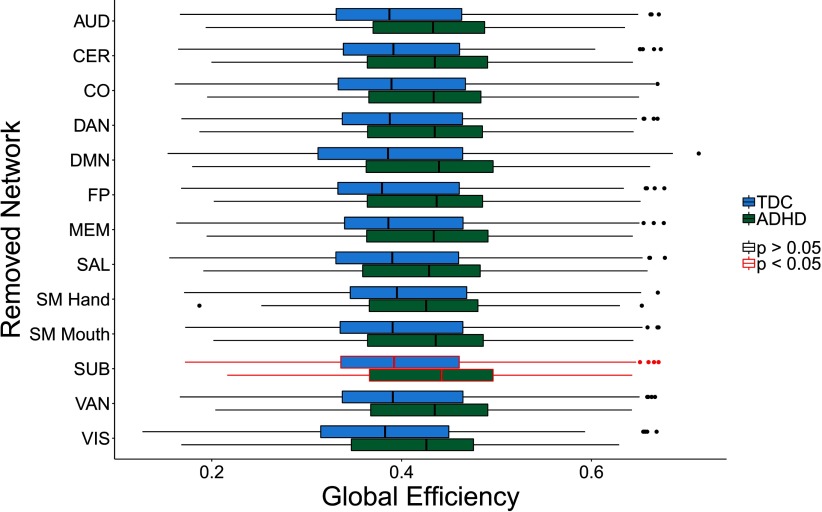
Group differences in global efficiency between children with ADHD and TD children after the removal of a given subnetwork. Networks are presented here in alphabetical order. Central line is the median, box is the interquartile range (IQR), and whiskers represent 1.5 *IQR. Points represent values more than 2 *SD* away from the mean. Removal of the SUB network resulted in children with ADHD having significantly higher global efficiency than TD children.

Removal of the SUB subnetwork led to a significant difference between children with ADHD and TD children with respect to global efficiency (Figure 4). Specifically, children with ADHD had greater global efficiency than TD children after SUB subnetwork removal. Table 1 in the Supporting Information contains the numerical differences and *p* values. It should be noted that these results are best interpreted when combined with the differential impact results below.

Differential impact of subnetwork removal: Finally, we examined whether the removal of any of the functional subnetworks led to a differential change in global efficiency for children with ADHD as compared with TD children.

**Figure 5.**
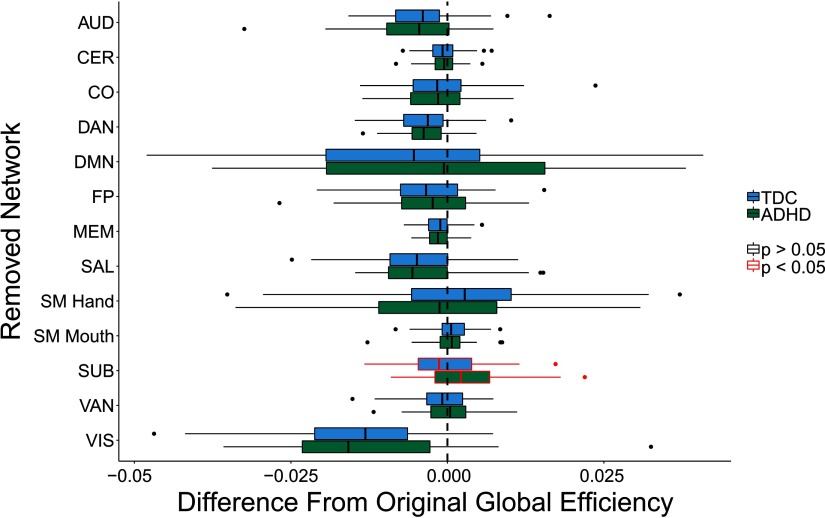
Differential impact on global efficiency when removing subnetworks between children with ADHD and TD children. Central line is the median, box is the interquartile range (IQR), and whiskers represent 1.5 * *IQR*. Points represent values more than 2 *SD* away from the mean. Removal of the subcortical subnetwork (SUB) led to a greater increase in global efficiency in children with ADHD as compared with TD children, suggesting that the SUB subnetwork is less integrated into the whole-brain network in children with ADHD. Significant differences were based on BH corrected *p* values.

The only subnetwork that exhibited significant differential impact after adjusting for multiple comparisons was the SUB subnetwork. Children with ADHD exhibited an increase in global efficiency after SUB subnetwork removal, while TD children exhibited a slight decrease (Figure 5). These findings suggest that the SUB subnetwork has lower integration with the global network in children with ADHD. The lower integration of the SUB subnetwork in children with ADHD is consistent with prior findings of disruptions in cortico-striatal-thalamic-cortical loops in ADHD (for a review, see Posner, Park, & Wang, 2014). Importantly, this finding is an example of equifinality, as the difference in the local topology of the subcortical network did not lead to an observed difference in whole-brain global efficiency. Table 3 in the Supporting Information contains the numerical differences and *p* values for this analysis.

## DISCUSSION

Whole-brain network analysis is a powerful tool for assessing brain topology, yet it lacks the ability to localize findings related to global topology to a specific location. This issue, combined with the fact that robust local differences do not necessarily result in observable global differences in topology, suggests that whole-brain network analysis has a substantial blind spot. Local graph theory analysis metrics can alleviate this but lack the ability to directly relate local findings to global topology. In this article, we introduced the NS jackknife, a flexible tool for localizing global differences in network topology. Our framework links the global scope to the local scope (Hallquist & Hillary, 2018) and is flexible enough to operate on any global network statistic. We implemented this framework in an open source R package, *netjack* (Henry, 2018; https://CRAN.R-project.org/package=netjack) to make it accessible to researchers. Our simulation study evaluating the performance of the NS jackknife showed that the method has excellent sensitivity to local differences in network topology across a variety of conditions, while keeping the false discovery rate at a nominal level (0.05; see the Supporting Information).

The NS jackknife is an extension of the simulated lesioning approach that offers a variety of advantages to neuroimaging researchers interested in examining group differences in network topology. The NS jackknife extends the simulated lesioning approach beyond its most common application, examining the robustness of a network to attack, thus enabling researchers to examine how individual elements of a given network contribute to a global network statistic. It can be applied to any global network statistic under study, which allows researchers to extend existing analysis pipelines rather than requiring implementation of a new set of analyses. The specificity of a global analysis can be tested at any level of resolution (subnetwork, node, edge), and resulting significance tests are appropriately corrected for multiple comparisons, making this an easy tool to implement and interpret. Finally, while the focus here was on binary networks derived from functional MRI data, the NS jackknife can easily be applied to weighted functional networks or to structural networks derived from diffusion-weighted imaging.

There are several further methodological developments for the NS jackknife. While we focused on comparisons between two groups of subjects, future versions of the NS jackknife toolbox will allow for within-subject across-time comparisons, testing covariates and controlling for potential confounds, and analyzing factorial grouping structures. The NS jackknife framework can be extended to generalized linear regression approaches as well as to allow for dependencies, such as those that arise from multiple collection sites. This is an active area of development, with implementations being introduced in the near future. Although the NS jackknife as currently presented examines network statistics as the outcome, it is likewise natural to consider network statistics as predictors of some distal outcome, such as behavior or symptomology. Our framework can be extended to handle cases where some distal outcome is of interest and would allow an assessment of the importance of local differences in network topology with regard to that distal outcome.

Finally, our empirical example produced results that were consistent with prior results suggesting little difference in global metrics of functional integration (i.e., global efficiency) between children with ADHD and TD children. Importantly, our use of the NS jackknife uncovered an occurrence of equifinality, in which a local group difference did not lead to a global group difference. Specifically, an analysis of group differences and differential impact of subnetwork removal showed that the subcortical subnetwork was less integrated into the whole-brain network in children with ADHD, consistent with prior literature (Hong et al., 2014).

### Limitations and Considerations

A key consideration for the use of the NS jackknife is in the use of weighted networks or networks formed from white matter tractography. The NS jackknife is agnostic to the type of network (weighted vs. binary, undirected vs. directed), but special care needs to be taken when using weighted networks to ensure that the appropriate network statistics are used. For example, when global efficiency is computed on correlation networks, it often exhibits scaling issues due to taking the inverse of correlations that are close to zero. If those correlations are removed during the NS jackknife, the resulting difference in global efficiency could simply be due to that scaling, and not due to any true difference in network structure. This is an issue for any weighted graph analysis and is not limited to the NS jackknife approach.

NS jackknife as a general framework has a limitation that users need to consider. As most tests of interest involve multiple comparisons, a multiple comparison correction is necessary to reduce the false discovery rate. Because of this, as the number of network features jackknifed increases, power to detect any given difference due to the removal of a single network feature decreases. This therefore limits the scope of an NS jackknife application. In smaller samples, NS jackknife is likely best applied to questions regarding functional subnetworks, in which the number of subnetworks is relatively small and potential impacts are large. For larger studies, particularly studies utilizing public datasets such as the Human Connectome Project (Van Essen et al., 2013) or the Autism Brain Imaging Data Exchange (ABIDE) datasets (Di Martino et al., 2017; Di Martino et al., 2014), it becomes more feasible to examine differences at the level of nodes or even edges. Another strategy that researchers with smaller samples can employ is examining more localized networks with the NS jackknife. For example, conducting an analysis limiting the network under consideration to the default mode network and removing nodes and edges of that smaller network rather than of the whole-brain network. This particular limitation, however, is inherent to neuroimaging analysis given the high-dimensional nature of the data. This speaks to a general need for the development of analytic methods of high-dimensional data dealing with issues of global to local scoping as discussed above. One potential solution to this problem is to use the screen filtering method of Meskaldji and colleagues (2015) to adjust the *p* values for tests of difference at the node or edge level with respect to subnetwork groupings.

For pedagogical purposes the manuscript focused on detecting local differences in single canonical functional subnetworks; however, the NS jackknife can be applied to combinations of canonical subnetworks as well combinations of nodes or edges. One key consideration here is that of combinatoric complexity. When using the NS jackknife to test removal of combinations of multiple subnetworks or nodes, the number of possible combinations increases factorially. To avoid this, the NS jackknife can be used in a confirmatory way, with researchers specifying a set of combinations of network structures informed by previous literature or a previous exploratory application of NS jackknife, then testing for local differences with that set of combinations. Finally, the NS jackknife does not require the use of canonical subnetworks as demonstrated here. Sample-specific parcellations derived from some other method can be used instead. The only requirement here is that a given network is defined on the same nodes for all subjects.

There are two additional considerations for researchers interested in using the NS jackknife framework. The first is that of network construction. The NS jackknife assumes a fixed network structure, and that the only changes that are occurring are due to the removal of specific network features. This means that network estimation techniques, such as regularized partial correlations, are to be used before the NS jackknife is applied, and not applied to the jackknifed networks themselves. This ensures that any localized effect can be interpreted as due to the removal of the specific structure only, and not an analytical artifact caused by a reestimation of the network.

The second consideration is that of the density/strength dependency. As mentioned earlier, network statistics (such as average degree) are dependent on the size and density of the network (van Wijk et al., 2010). As such, it is expected that the network statistics would change when structures are removed. This potentially causes issues with interpretation, where an observed group difference might simply be due to differences in density of the removed component rather than any topological difference. One potential solution to this interpretational concern is to add the network element’s density or strength as a covariate to the jackknife models. Research into adjustments for density/strength dependency is ongoing.

## CONCLUSION

In closing, the NS jackknife framework bridges the gap between global network analysis and local differences in topology, making it an ideal tool with which to investigate the specificity of observed differences in whole-brain topology. Furthermore, it allows researchers to explore local network features in the absence of a global result while still using the tools, terminology, and interpretation of network neuroscience at the global level. Finally, future developments will extend the NS jackknife to more complex modeling frameworks and settings, further extending its utility within the neuroimaging community.

## SUPPORTING INFORMATION

Supporting information for this article is available at https://doi.org/10.1162/netn_a_00109. The NS jackknife method is available at https://cran.r-project.org/package=netjack (Henry, 2018).

## AUTHOR CONTRIBUTIONS

Teague Henry: Formal analysis; Investigation; Methodology; Software; Visualization; Writing – Original Draft; Writing – Review & Editing. Kelly A. Duffy: Data curation; Resources; Writing – Review & Editing. Marc D. Rudolph: Writing – Original Draft; Writing – Review & Editing. Mary Beth Nebel: Data curation; Writing – Review & Editing. Stewart H. Mostofsky: Data curation; Funding acquisition; Writing – Review & Editing. Jessica R. Cohen: Conceptualization; Funding acquisition; Resources; Supervision; Writing - Original Draft; Writing – Review & Editing.

## FUNDING INFORMATION

Jessica R. Cohen, National Institute of Mental Health (US), Award ID: R00MH102349. Stewart H. Mostofsky, National Institute of Mental Health (US), Award ID: R01MH085328. Stewart H. Mostofsky, National Institute of Mental Health (US), Award ID: R01MH078160. Mary Beth Nebel, National Institute of Mental Health (US), Award ID: K01MH109766.

## TECHNICAL TERMS

Jackknife: A nonparametric statistical technique that involves repeatedly reestimating a parameter value after the removal of single observations.
Whole-brain (global) network statistic: A statistic derived from a whole-brain network that describes an aspect of global topology.
Global topology: The overall shape and structure of a network.
Specificity: When differences in global topology are being driven by specific differences in local topology, as opposed to globally distributed differences.
Equifinality: When different network configurations can lead to the same global network statistic.
Virtual/simulated lesioning: A technique in neuroimaging that removes components of a network during analysis to examine the impact of those components on global topology.
Network robustness: How much a network’s global topology changes when nodes/edges are removed. This removal is typically done in a random or semirandom fashion to assess the network’s robustness to attack or damage.
