# Supplementary material for: Bridging global and local topology in whole-brain networks using the network statistic jackknife
Source: Netw Neurosci. 2020 Feb 1;4(1):70–88. doi: 10.1162/netn_a_00109 (PMC7006875; doi:10.1162/netn_a_00109)
Supplement: Supplementary file 1 [file netn-04-70-s001.pdf]

Henry, T. R., Duffy, K. A., Rudolph, M. D., Nebel, M. B., Mostofsky, S. H., Cohen, J. R. (2020). Supporting information for "Bridging Global and Local Topology in Whole-brain Networks using the Network Statistic Jackknife." *Network Neuroscience*, *4*(1), 70-88. https://doi.org/10.1162/netn_a_00109

Supplementary Materials

Table 1: Group Differences in Global Efficiency.

Removed Subnetwork	Mean for ADHD	Mean for TDC	p-value
Auditory	0.425833	0.394577	0.087098
Cerebellar	0.429974	0.398176	0.080539
Cingulo-Opercular	0.429001	0.397225	0.08205
DMN	0.429129	0.393425	0.081011
Dorsal Att	0.427206	0.395244	0.083384
Fronto-Parietal	0.427893	0.395041	0.075489
Memory	0.429311	0.39744	0.083003
Salience	0.426803	0.393741	0.077377
SM Hand	0.428592	0.40114	0.121957
SM Mouth	0.430966	0.399428	0.082671
SubCortical	0.43374	0.398171	0.048334
Ventral Att	0.430935	0.398113	0.072409
Visual	0.417344	0.384474	0.090177

Table 3: Differential Impact in Global Efficiency.

Removed Subnetwork	Mean Difference for ADHD	Mean Difference for TDC	p-value	BH Adjusted p-value
Auditory	-0.00472	-0.00417	0.607595	0.912567
Cerebellar	-0.00058	-0.00057	0.99822	0.99822
Cingulo-Opercular	-0.00155	-0.00152	0.981411	0.99822
DMN	-0.00142	-0.00533	0.263888	0.785685
Dorsal Att	-0.00334	-0.00351	0.827491	0.99822
Fronto-Parietal	-0.00266	-0.00371	0.392448	0.850303
Memory	-0.00124	-0.00131	0.867592	0.99822
Salience	-0.00375	-0.00501	0.302187	0.785685
SM Hand	-0.00196	0.00239	0.08175	0.531376
SM Mouth	0.000417	0.000678	0.631777	0.912567
SubCortical	0.003191	-0.00058	0.000473	0.006143
Ventral Att	0.000386	-0.00064	0.164322	0.712061
Visual	-0.0132	-0.01428	0.599352	0.912567

Simulation Methods and Results

Generative Model: The present study uses the stochastic block model of Nowicki and Snjiders (2001) to generate the networks for analysis. This model proposes that for a network G comprised of n nodes, the edge between nodes i and j is distributed as a

Bernoulli random variable. The probability of an edge being present is a function of the community membership of nodes i and j. For example, the probability of an edge between two nodes in the same community could be set to .8, while the probability of an edge between two nodes in different communities could be set to .05, which would result in an extremely modular network. This model creates a binary, modular network and can be extended to any size of network. Additionally, as the network is defined at the subnetwork level it is particularly relevant to the NS jackknife, as removal of subnetworks to detect group differences is one of the main usages of this approach.

Simulation Design: This simulation study was designed to evaluate the performance of the NS Jackknife under several instances of group differences. To achieve this, within each of the below conditions we defined two generative models, Group 1 and Group 2. From those generative models, we simulated an equal number of networks, which form the samples of networks for Groups 1 and 2. This is analogous to constructing networks from two groups of subjects' resting state fMRI timeseries. For all networks, we defined a priori five subnetworks of equal size. Equal community sizes were chosen for two reasons: 1) this allowed for better control of the precise nature of differences between the groups; 2) the impact of subnetwork size is being evaluated by changing the total network size in the simulations. Within each condition, sample size, network size and group difference combination, 250 iterations were performed.

Conditions:

Overall Design Factors – Across all generative models, we evaluated the effect of sample size and network size. Sample size ranged from 10 to 100 in each group in steps of 10, while network size ranged from 50 to 300 nodes in steps of 50. These values were chosen to correspond to reasonable sample sizes and network sizes in neuroimaging applications. Condition 1 had no group difference, while for conditions 2 through 4 we manipulated the level of group difference as low or high.

For Conditions 2, 3 and 4, the level of group difference was also manipulated. We defined the low group difference as a relative difference in probability between groups of .01, while for the high group difference this was .1. For example, Condition 2 examines within community differences, so for the low group difference iterations, the relative within community probabilities was set to .31 for a relevant group (while the other group was set to probability .3), and for the high group difference iterations the relevant probabilities for the relevant group was set to .4 (while the other group was set to .3).

Condition 1: No Group Difference – This was our no-effect condition. As such, the generative models for Groups 1 and 2 were identical. The within community probability was set to .3 for all communities, and the between community probability was set to .1 between all pairs of communities. The Condition 1 model was our base model, from which all other conditions differed in specific ways. Figure 1 (1st row) shows an example adjacency matrices generated from this model.

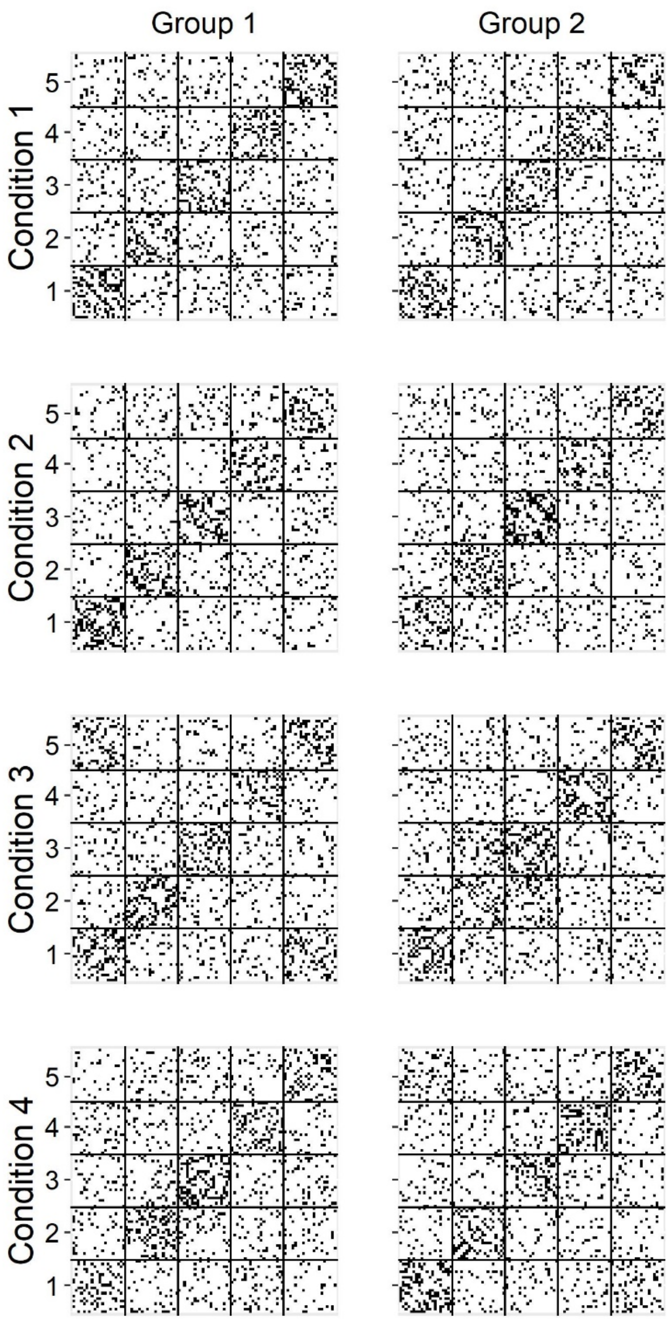

Figure 1: Example adjacency matrices generated from all conditions and all groups. 100 nodes, with 5 subnetworks/communities of 20 nodes each. Black squares represent the presence of an edge. Black lines subdivide the network into the 5 communities. The probability of an edge within any community is .3, while the probability of an edge between two communities is .1.

Condition 2: Within Community Equifinality – This condition consisted of groups with equivalent whole-brain global network statistics, but used differences in within community probability to detect differences localized to specific subnetworks. We began

with the same generative model used in Condition 1, with the following changes. For the low group difference condition, the probability of having an edge within subnetwork 1 was increased to .31 for Group 1 and remained .3 for Group 2, while the probability of having an edge within subnetwork 3 remained .3 for Group 1 and was increased to .31 for Group 2. In the high group difference condition, the probability of having an edge within subnetwork 1 was increased to .4 for Group 1 and remained .3 for Group 2, while the probability of having an edge within subnetwork 3 remained .3 for Group 1 and was increased to .4 for Group 2. This mirrored difference, in which each group differs from each other but in the same fashion, allows the global network statistics (density, modularity, global efficiency) to have the same expected value for each group. Figure 1 (2nd row) presents an example adjacency matrix from Condition 2.

Condition 3: Between Community Equifinality – This condition was similar to Condition 2 in that it used differences in the generative model that resulted in the same overall whole-brain global network statistics for each group. However, the changed probability was the between community probability instead of the within community probability. Again, we began with the same generative model used in Condition 1, with the following changes. For the low group difference condition, the probability of having an edge between nodes in communities 1 and 5 was increased to .11 for Group 1 and remained .1 for Group 2, while the probability of having an edge between nodes in communities 2 and 3 remained .1 for Group 1 and was increased to .11 for Group 2. For the high group difference condition, the probability of having an edge between nodes in communities 1 and 5 was increased to .2 for Group 1 and remained .1 for Group 2, while the probability of having an edge between nodes in communities 2 and 3 remained .1 for Group 1 and was increased to .2 for Group 2. Figure 1 (3rd row) shows an example adjacency matrix from Condition 3.

Condition 4: Specificity – This condition evaluated group differences in network structure that would result in a difference in the whole-brain global network statistics. Here, Group 1 had the Condition 1 generative model, while Group 2 had the same generative model as Group 1 in Condition 3. Group 2 therefore began with the same generative model with the following changes: an increased probability of an edge between communities 1 and 5 from .1 to .11 for the high group difference condition and to .2 for the high group difference condition. Figure 1 (4th row) contains an example of an adjacency matrices from Condition 4.

Analysis:

We applied the NS jackknife to localize group differences in both global efficiency and modularity. As the results for global efficiency and modularity were similar, only modularity results are presented here. The jackknife targets were the 5 communities, and both the group test and the differential impact test were performed. For Condition 1 the relevant outcome is the Type I error rate, as Groups 1 and 2 do not differ in that condition. For Conditions 2 through 4 the outcome is power, specifically to detect different impacts on the whole-brain statistic as related to the relevant subnetworks. Additionally, we examine the effect size of the differences between subnetworks.

All code used in this simulation has been stored on the manuscripts OSF site (https://osf.io/ecvx7/)

For Condition 1 the relevant outcome is the Type I error rate, as Groups 1 and 2 do not differ in that condition. For Conditions 2 through 4, the outcome is power, specifically to detect the differences in the relevant subnetworks. Additionally, we examine the absolute group differences in for each jackknifed component.

Results:

Condition 1 - No Group Difference – Figure 2 presents the false positive rate and absolute group differences for Condition 1 across sample size, network size for the NS jackknife.

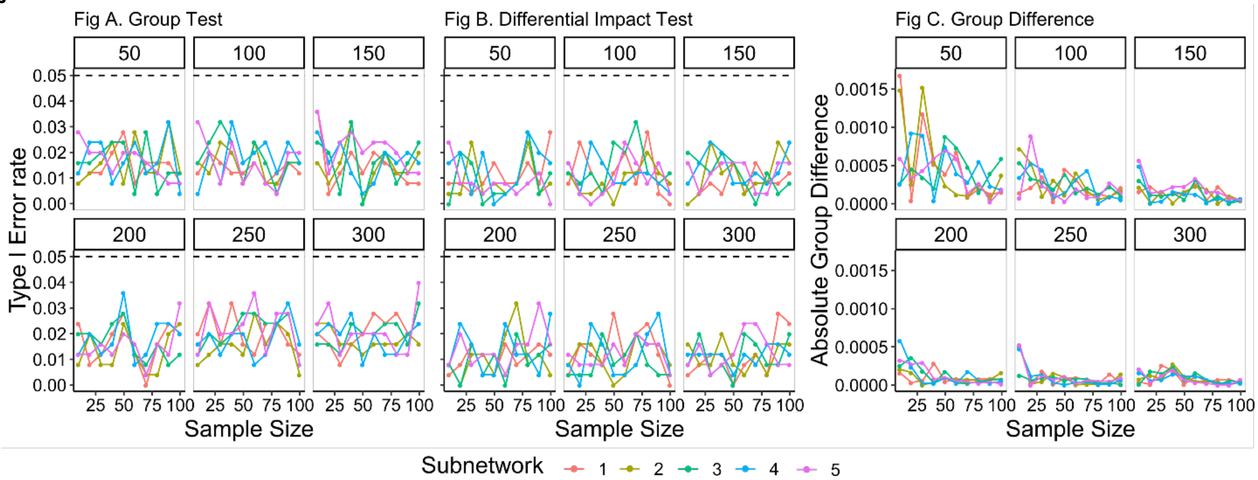

Figure 2: Condition 1 – No Group Difference. Type I Error rate for the NS Jackknife for the Group Test (1st figure), Differential Impact Test (2nd figure). The third figure contains the absolute group differences. Design factors were sample size (x axis), network size (panel) and subnetwork (color). The dotted line is at .05. Note that for all subnetworks, under all conditions, the Type I Error rate does not exceed .05 and is, on average, around .02.

The results presented in Figures 2 show that the NS Jackknife properly controls for Type I error rate, and is well below the nominal alpha of .05 across all conditions for both the Group test and the Differential Impact test. Additionally, the variability in the absolute group difference decreases as a function of network size and sample size.

Condition 2 – Within Network Equifinality. This condition evaluates the ability of the NS Jackknife to detect local differences in the absence of a global difference when they occur within a community. The two subnetworks that differ between groups 1 and 2 are that of subnetwork 1 and subnetwork 3. Overall, global modularity does not differ between the groups. This condition has two group difference sizes, low and high. Figures 3 presents the power and absolute group difference for the Group Test and

Differential Impact Test for the low and high group difference analysis (top panel and bottom row respectively).

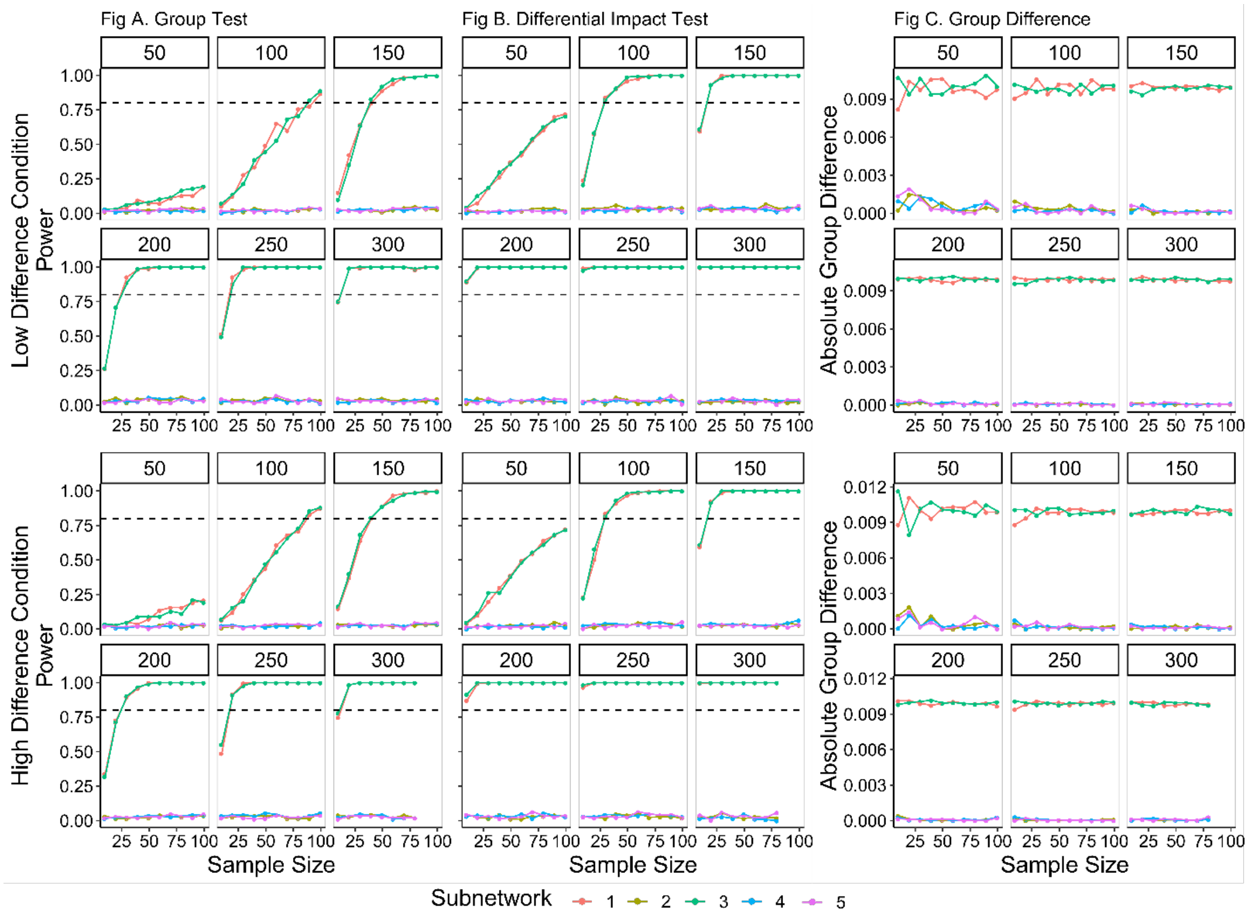

Figure 3: Condition 2 – Within Community Equifinality. Power and Absolute Group Difference for the NS Jackknife Group Test by sample size (y axis), network size (panel), group difference (row) and subnetwork (color). The dotted line is at .8. Results suggest good power to identify the relevant subnetworks (1 and 3) when network sizes are moderate (150+) and sample size is moderate (40+ in each group). High group difference increases power across all conditions. Notably, network size moderates the effect of sample size on power in all conditions, with larger network sizes leading to a sharper increase in power with sample size. Finally, this condition demonstrated good specificity, in that it did not detect differences in the non-relevant subnetworks.

The results from the high group difference condition were extremely similar to that of the low group difference condition. The power of the Group Test was slightly increased, while the power of the differential impact test did not appear to be affected by the increase in group difference. This is likely due to modularity's relative lack of sensitivity to within module changes.

Condition 3 – Between Subnetwork Equifinality:
Condition 3 evaluated the ability of the NS Jackknife to detect group differences in between community edge probabilities. The relevant subnetworks are 1, 2, 3 and 5, while 4 should not differ between groups. Like Condition 2, the groups have equivalent global modularity values. In the low group difference condition, the differences between the relevant edge probabilities is .01 (.1 vs .11) and for the high group difference .1 (.1 vs. .2). Figure 4 presents the results from this condition.

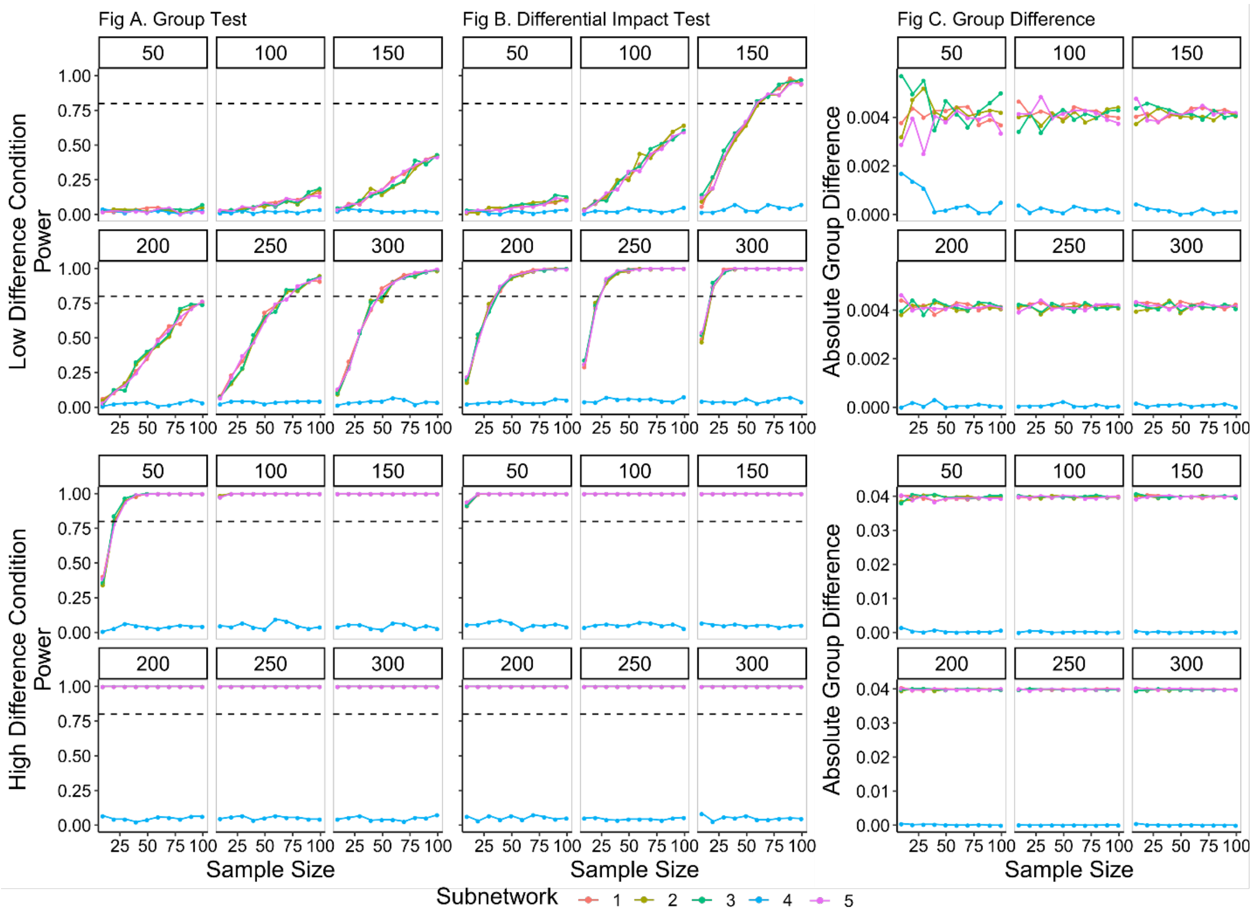

Figure 4: Condition 3 - Between Subnetwork Equifinality. Power and Absolute Group Difference for the NS Jackknife Group Test by sample size (y axis), network size (panel), group difference (row) and subnetwork (color). The dotted line is at .8. Results suggest extremely low power to detect the low group difference in small networks, while power increases with network size and sample size. This relation is less strong than in Condition 2. In the high group difference conditions, power is extremely good, even for small networks (50+ nodes) and small sample sizes (20+ subjects in each group). As in Condition 2, the differential impact test has greater power than the group test in all conditions. Specificity is excellent across all conditions as well. The strong effect of the high group difference is likely due to modularity’s reliance on between subnetwork connections.

Condition 4 – **Specificity:** Condition 4 tests the ability of the NS Jackknife and screen filtering method to localize differences in modularity when global differences in modularity are present. All subnetworks are relevant, due to the differences in global modularity, but the most relevant subnetworks are 1 and 5. The low group difference condition puts the probability of an edge between 1 and 5 in Group 2 to .11 (vs. .1 in Group 1), while the high group difference condition puts that probability at .2. Figure 5 presents the findings from this condition.

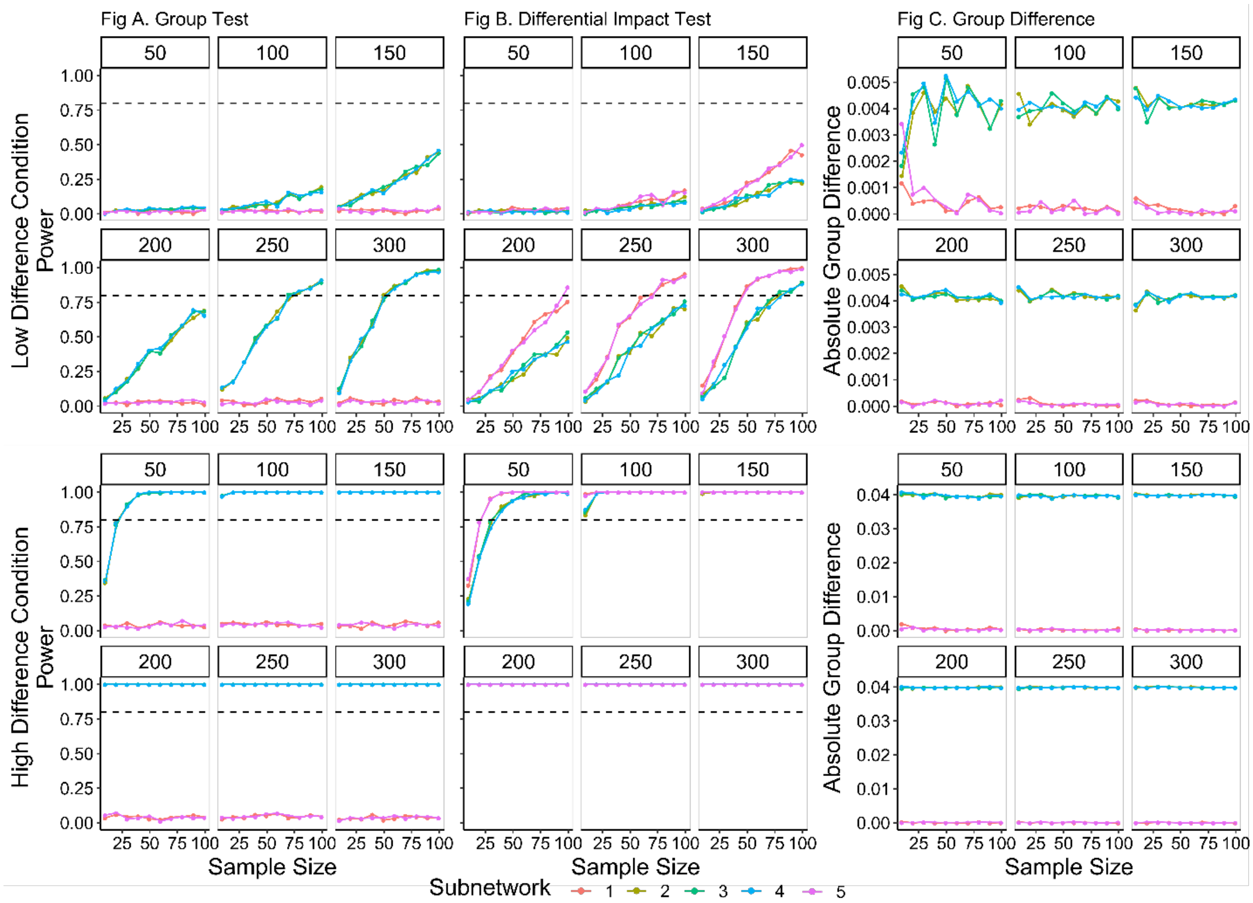

Figure 5: Condition 4 - Specificity. Power and Absolute Group Difference for the NS Jackknife Group Test by sample size (y axis), network size (panel), group difference (row) and subnetwork (color). The dotted line is at .8. These results indicate that the removal of any subnetwork *except* networks 1 and 5 result in a significant difference between groups, while when subnetwork 1 or 5 are removed, there is no significant difference between groups. Furthermore, the power to detect differences when subgroups 2, 3 or 4 are removed show the expected relation to network size/sample size, while there is no change in the ability to detect differences in subnetworks 1 or 5. The Differential Impact Test shows the expected relation with sample size and network size to detect differences in all subnetworks, but the power increases more quickly for subnetworks 1 and 5. The Absolute Group Differences indicate the the removal of either subnetwork 1 or 5 results in no group difference between the groups. This follows from the generating model, as the removal of either of those subnetworks result in networks that are identical across groups.

The results for Condition 4 suggest that when there exists a difference in the global network statistic, the NS jackknife can localize the difference. However, care must be taken, as non-significant group differences may correspond to the most important contributors to the global difference in the network statistic. This in turn suggests that when a global difference exists, researchers need to examine the group test, differential impact test, and effect sizes for both the group difference and the differential impact test (not shown here), to help disentangle the correct effect.

The high group difference condition shows that with increases in the difference of the global network statistic between groups, the NS Jackknife is increasingly able to detect that the networks are different in some way. The core concern here is that the NS Jackknife does not directly distinguish which subnetworks are most relevant to the global group difference. This could be inferred by examining both the group test and the differential impact test. When the group test shows no difference when a sub network is removed, but does show differential impact of that subnetwork, this suggests that that specific subnetwork is a major contributor to the differences in the global network statistic. Furthermore, one can examine the effect sizes of the differential impact test. Figure 6 shows an example of that applied to the high group difference condition of Condition 4.

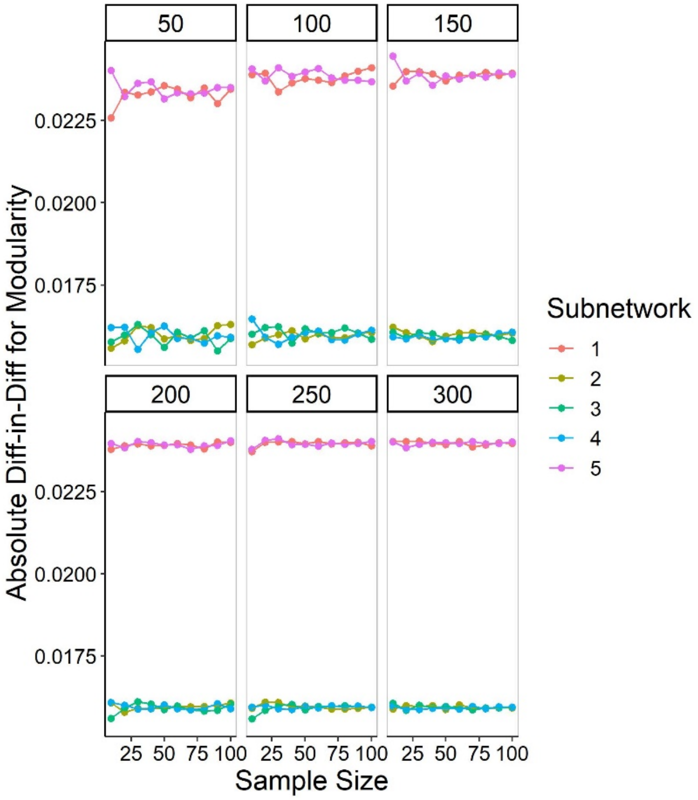

Figure 6: Condition 4 (High Group Difference). Absolute Difference-in-Difference in modularity by sample size (y axis), network size (panel) and subnetwork (color). Results

show that subnetworks 1 and 5 are correctly identified as the largest contributors to the difference in the global modularity.

Summary: This simulation study suggest that the NS jackknife is a powerful tool for localizing whole brain network statistics, but that this power comes with several interpretational complications. In the case that the target whole brain network statistic is not significantly different between groups, the NS jackknife can identify which subnetworks differ between groups with respect to their contribution to the global network statistic.

However, in the case of a preexisting difference in global network statistics, the interpretation of the NS jackknife becomes more difficult. Instead of interpreting the group test and differential impact test separately, they must be interpreted together. A non-significant group test with a significant differential impact test suggest that the removal of that particular network element leads to there being no difference between groups, which in turn suggest that the given network element is central to those group differences. Fortunately, this can be detected by examining effect sizes, particularly that of the differential impact test.

In terms of power and Type I error rate, the network size interacts with sample size to increase the power to detect small effects. This suggests that a large network combined with a large sample size would be ideal for detecting differences in subnetworks. These results also extend to cases where there are more subnetworks, or the subnetworks are of differing size. In all cases, the power of the NS jackknife will be determined by the amount of information a given network component contains about the network statistic in question. In a network with many subnetworks, the removal of a small subnetwork (for example, 5 nodes) will result in a less powerful test than the removal of a large subnetwork (such as the DMN, which often contains 30+ nodes in most functional atlases). However, control over the false discovery rate is maintained, regardless of subnetwork size, due to the strong control applied by the BH correction.

Limitations: There are several limitations to this simulation study. The first is that the generative models used are one type of network model out of many. With different network generating model, it is likely one would see different patterns of power and false positive rates, however, the presented results make a strong argument that the direction of the associations would be the same (i.e. as network size/sample size increase, the power of the NS jackknife increases). Additionally, only one network statistic was assessed here. Modularity is ideal for examining networks with clear subnetwork structure, but the specific choice of a network statistic will be determined by a given study. Some network statistics might lead to more or less reliable results. One immediate example is that of global efficiency computed on correlation networks, as this often results in numeric instability and scaling issues due to the presence of values extremely close to 0. This instability would likely lead to problems in analysis using the NS jackknife. We caution researchers to use network statistics that are 1) appropriate to the type of network at hand (undirected/directed, unweighted/weighted) and that are directly relevant to a given research question.

References:

Benjamini, Y., & Hochberg, Y. (1995). Controlling the False Discovery Rate: A Practical and Powerful Approach to Multiple Testing. *Journal of the Royal Statistical Society. Series B (Methodological)*, *57*(1), 289–300. https://doi.org/10.2307/2346101

Nowicki, K., & Snijders, T. A. B. (2001). Estimation and Prediction for Stochastic Blockstructures. *Journal of the American Statistical Association*, *96*(455), 1077–1087. https://doi.org/10.1198/016214501753208735

Meskaldji, D. E., Vasung, L., Romascano, D., Thiran, J. P., Hagmann, P., Morgenthaler, S., & Van De Ville, D. (2015). Improved statistical evaluation of group differences in connectomes by screening-filtering strategy with application to study maturation of brain connections between childhood and adolescence. *NeuroImage*, *108*, 251–264. https://doi.org/10.1016/j.neuroimage.2014.11.059
